# Sleep/Wake Regularity Associated with Default Mode Network Structure among Healthy Adolescents and Young Adults

**DOI:** 10.1038/s41598-019-57024-3

**Published:** 2020-01-16

**Authors:** Jessica R. Lunsford-Avery, Katherine S. F. Damme, Matthew M. Engelhard, Scott H. Kollins, Vijay A. Mittal

**Affiliations:** 1 Department of Psychiatry and Behavioral Sciences, Duke University School of Medicine, Durham, North Carolina USA; 2 Department of Psychology, Northwestern University, Evanston, IL USA

**Keywords:** Sleep disorders, Risk factors

## Abstract

Sleep deprivation and disorders are linked to reduced DMN connectivity. Less is known about how naturalistic sleep patterns – specifically sleep irregularity - relate to the DMN, particularly among adolescents and young adults. Additionally, no studies have utilized graph theory analysis to clarify whether sleep-related decreases in connectivity reflect global or local DMN changes. Twenty-five healthy adolescents and young adults (age range = 12–22; mean = 18.08; SD = 2.64, 56% female) completed 7 days of actigraphy and resting-state fMRI. Sleep regularity was captured by the Sleep Regularity Index (SRI) and the relationship between the SRI and DMN was examined using graph theory analysis. Analogous analyses explored relationships between the SRI and additional resting-state networks. Greater sleep regularity related to decreased path length (increased network connectivity) in DMN regions, particularly the right and left lateral parietal lobule, and the Language Network, including the left inferior frontal gyrus and the left posterior superior frontal gyrus. Findings were robust to covariates including sex and age. Sleep and DMN function may be tightly linked during adolescence and young adulthood, and reduced DMN connectivity may reflect local changes within the network. Future studies should assess how this relationship impacts cognitive development and neuropsychiatric outcomes in this age group.

## Introduction

Sleep is integral to brain functioning and cognition^1^. In the last decade, there has been growing interest in the impact of sleep on the spontaneous neuronal activity of the brain as assessed by resting-state functional connectivity magnetic resonance imaging^2^. In particular, of the six identified resting-state networks, particular attention has been paid to the role sleep may play in the functional connectivity of the default mode network (DMN)^3^ – and to a lesser extent, its anticorrelated network (ACN) – given the strong associations of both sleep and these specific networks to cognitive function^4–8^.

The DMN spans a network of brain regions – including the bilateral inferior parietal, medial prefrontal, retrosplenial, and posterior cingulate cortices, precuneus, and portions of the hippocampus and medial temporal lobes – and is closely tied to cognition^9^. Specifically, the DMN deactivates during externally-oriented, goal-directed tasks^9–11^ and activates during internally-oriented, self-referential tasks, such as episodic/autobiographical memory retrieval, daydreaming, and envisioning the future^10,12–15^. Reduced DMN functional connectivity is implicated in a range of psychiatric and neurological conditions impacting cognition across the lifespan, including attention-deficit/hyperactivity disorder^16^, schizophrenia^17^, depression, and dementia^18^ - disorders also associated with pervasive sleep complaints^19^.

To date, research investigating links between sleep and DMN functional connectivity has largely consisted of experimental sleep deprivation studies or examinations of sleep-disordered populations rather than naturalistic observations of sleep behavior. Specifically, prior work has shown that sleep deprivation results in reduced functional connectivity of the DMN and the DMN-ACN anticorrelation during both rest and task performance and among both young and older adults^4,6^. In addition, reduced DMN connectivity has been observed among young and elderly adults who report high levels of daytime sleepiness^7^ as well as individuals diagnosed with insomnia and obstructive sleep apnea (OSA) disorders^3,5,20^. Taken together, these studies suggest a close association between sleep and DMN connectivity and highlight a potential neural mechanism by which poor sleep results in cognitive deficits.

Less is known about how another important aspect of sleep – the regularity of sleep/wake patterns – is related to resting-state DMN connectivity. Sleep regularity, as calculated by the Sleep Regularity Index (SRI) and related measures, has been shown to be associated with cognitive performance^21^, academic performance^22^, and health outcomes^23^ across the lifespan. In addition, most research to date has focused on examining the impact of sleep on resting-state DMN connectivity in adulthood. In contrast, very little is known about the potential effect of sleep on DMN connectivity during adolescence, a developmental period characterized by significant shifts in both sleep behavior/physiology as well as brain maturation and functioning^24^.

Investigating the impact of sleep regularity and resting-state DMN connectivity during adolescence is important for several reasons. First, studies have shown that sleep patterns normatively become less regular across adolescence and into early adulthood^25–28^, suggesting this period is particularly critical for the development of sleep pattern regularity. Moreover, irregularity in sleep patterns among adolescents has been shown to be related to altered brain development, such as reduced white matter integrity, over and above the impact of shortened sleep duration^29^, as well as reduced activation of the medial prefrontal cortex and striatum in response to reward^30^. These studies suggest that sleep regularity may be vital to brain development and function during adolescence.

Second, the DMN matures significantly during adolescence. For example, within-network connectivity of the DMN increases between ages 10 and 13; simultaneously between-network segregation between the DMN and central executive network (a network that includes dorsal frontal and parietal regions) increases^31^. In another example of age-based changes over development, deactivation of the DMN becomes increased and more distributed during cognitive task performance in older adolescents compared to younger ones^32^. Finally, across development from childhood to adulthood, DMN resting-state connectivity shows increased integration and inter-network segregation^33,34^, both of which are associated with the development of complex cognition^35^.

To our knowledge, only one prior study has examined a potential relationship between naturalistic sleep and DMN connectivity among adolescents (aged 14–18), and found that poorer sleep quality (a composite variable encompassing actigraphically-measured sleep efficiency, number of awakenings, and duration of awakenings), but not sleep duration, was correlated with weaker DMN connectivity^36^. This result suggests that aspects of naturalistic sleep may be tied to DMN functioning during this critical developmental period. The current investigation builds on this prior study by utilizing a recently developed and highly sensitive measure – the SRI – to examine how naturalistic patterns in sleep regularity may contribute to DMN structure. The SRI has several advantages over other measures of sleep regularity in that it does not require a primary sleep period (capturing napping behavior) and uses a day-to-day timescale that allows for assessment of rapid changes in sleep/wake patterns^22^.

Furthermore, although many studies examine DMN connectivity, fewer studies have examined features of the network structure using graph theory analyses. This innovative approach includes several metrics that may provide unique insight into the way in which connectivity changes. While previous studies focused on a global metric of correlations between network seeds, graph theory provides the ability to ask more intricate questions about the nature of dynamic network changes^37,38^. The clustering coefficient provides information about whether observed changes in connectivity are driven by changes in dynamic relationships (clustering) between regions whereas the path length metric allows for analysis of the connectivity between regions by way of other regions. For example, graph theory metrics clarify whether previously reported relationships between sleep quality and decreased DMN connectivity reflect global changes to the network or local changes in specific brain regions within the network.

The current study aimed to assess the relationship of sleep regularity, as measured by the SRI, to resting-state networks among adolescents and young adults. Based on the past literature, we hypothesized that regular sleep/wake patterns would be associated with improved network structural efficiency (i.e., decreased path length and increased clustering coefficient) within the DMN. We also investigated whether the relationship between increased sleep regularity and resting-state efficiency would be specific to the DMN, rather than resulting in a general benefit across all networks. To investigate this possibility, we examined the relationship between sleep regularity and resting-state network structures, including language, salience, dorsal attentional, sensory-motor, and frontal-parietal networks^39^. In addition to exploring the specificity of this effect to the DMN, this approach has the benefit of examining the understudied possibility that increased sleep regularity could benefit additional resting-state networks. Finally, the current study examines local graph metrics, which may highlight whether network organization surrounding particular regions within networks relates to sleep regularity.

## Methods

### Participants and procedure

A total of 25 healthy participants (age 12–22, mean = 18.08, SD = 2.64; 14 (56%) females) were recruited through the Adolescent Development and Preventive Treatment (ADAPT) Program via media announcements. The sample was 52% white, 32% Hispanic, and 16% Asian, which is more diverse than the population of Boulder, Colorado and the surrounding areas, and the mean parental education level (a socioeconomic proxy) was 16.28 years (SD = 2.40). The ADAPT program examines biological factors contributing to the development of typically-developing adolescents as well as those with psychiatric conditions, and participants were self-referred for participation in this study. The age range in the current study was selected based on literature suggesting that shifts in the regularity of sleep patterns begin during early adolescence and persist into early adulthood^25–28^. Participants (and for those under age 18, their parent or legal guardian) completed informed consent/assent. All procedures were approved by the institutional review board of the University of Colorado Boulder and all research was performed in accordance with relevant guidelines and regulations. During an intake visit, participants (and if <18 years old, their parent/guardian) completed a clinical interview (i.e., Structured Clinical Interview for the DSM-IV (SCID), research version^40^) and a medical history to rule out psychiatric, substance use, medical, and neurological disorders. All participants were screened to ensure no metal placement in the body. Of 33 possible participants, 25 met all inclusion criteria (including actigraphy criterion described below) and participated in the current study. The majority of the sample (92%) were right handed, but removing left handed individuals from the analyses (*n* = 2), did not impact the magnitude or the direction of the findings.

### Actigraphy

Sleep regularity was assessed via an Acti-Sleep monitor (Pensacola, FL) worn continuously on the participants’ non-dominant wrist over a 7 day period. Participants also completed a daily paper sleep/wake diary, which recorded bed-, wake-, and naptimes as well as nighttime awakenings and times the actigraph was removed. Data were collected in 60-second epochs and each epoch was labelled as sleep or wake using a clinically-validated decision tree algorithm^41,42^. Data were visually inspected for accuracy using the sleep/wake diary. Participants were required to have at least 5 valid days of actigraphy recording for inclusion in analyses. The SRI is defined as “the likelihood that any two time-points (minute-by-minute) 24 hours apart were the same sleep/wake state, across all days”^22^ and was calculated as previously reported by our group and others^22,23,43^, details of which are provided in our prior publication^23^. SRI scores range from 0–100. For analyses, SRI scores were converted to z-scores as mean-centering covariates has been found to be an effective way to reduce co-linearity and increase power for detecting signal in resting-state functional connectivity^44^. As such, SRI z-scores were used to maximize the sensitivity to signal by reducing co-linearity of signal. Higher scores indicated greater sleep regularity.

### MRI acquisition and processing

A 3T Siemens Tim Trio MRI Scanner (Siemens AG, Munich, Germany) acquired images with a standard 12-channel head coil. Images included a structural/T1 image for registration and a resting-state functional image. The structural/T1-weighted image was acquired in the sagittal plane and utilized a magnetization prepared rapid gradient multi-echo (MPRAGE) approach (192 interleaved slices; 256 mm field of view; isotropic voxels 1 mm^3^, GRAPPA parallel imaging factor of 2, Time to repetition (TR) = 2.530 s; Times to Echo (TE) = 1.64 ms, 3.5 ms, 5.36 ms, 7.22 ms, 9.08 ms; flip angle = 7°). A resting-state blood oxygen level dependent (BOLD) scan was acquired with a T2*-weighted echo-planar functional protocol (33 slices in a 240 mm field of view; 3.8 × 3.8 × 3.5 mm voxels; TR = 2.00 s; TE = 29 ms; Flip Angle = 75°). Participants were instructed to close their eyes during the resting-state scans which lasted 5 min 34 s.

### Resting-state data processing

FSL v6^45^ was used to preprocess data and these steps included a 100 s high-pass filtering and 6-mm FWHM kernel spatial smoothing after brain extraction. Resting state images were first aligned to the native space structural image, then to a template space (Montreal Neurological Institute 2-mm brain template) in a two-step process. Temporal derivative regressors were used to account for motion and were calculated using the artifact detection software (ART^46–49^). The ART software calculated three translation and three rotation parameters, as well as a series of image specific confound regressors which noted outliers in framewise movement and brain activation. To identify brain activation outliers, voxel was compared based on the z-normalized mean signal across all voxels as a function of time and were considered outliers if they exceeded a z-score of 3. A composite measure of total motion, or maximum voxel displacement, across translation and rotation identified any motion outliers as frames where the absolute value of motion exceeded 1 mm. All motion regressors were defined at the subject level of the model as a temporal derivative nuisance covariate.

### Connectivity matrices

**Figure 1:**
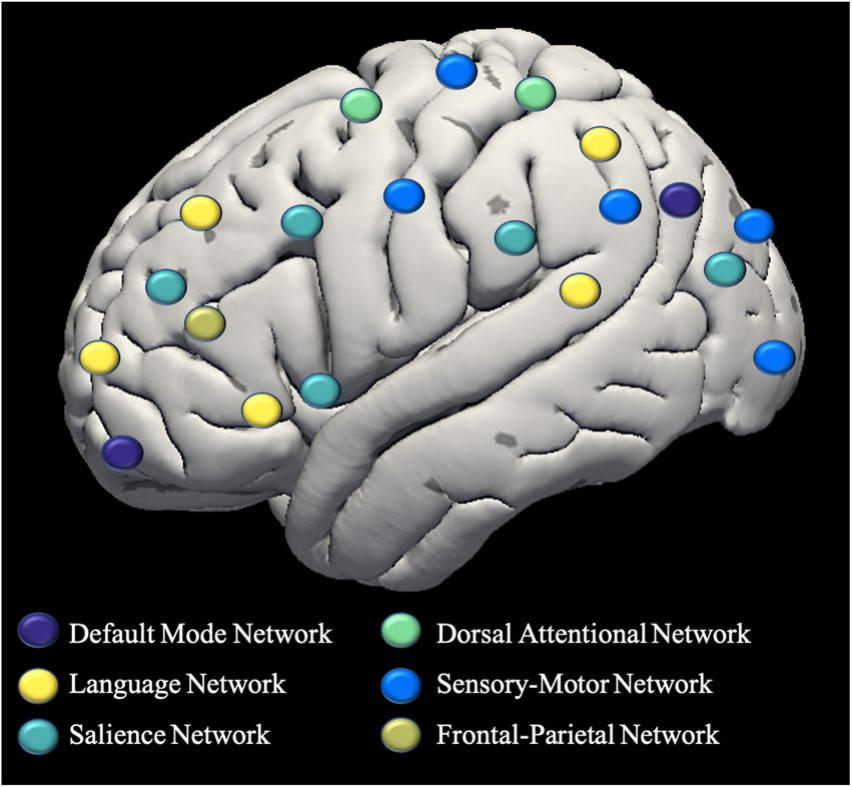
Network Atlas.

CONN toolbox ROI-to-ROI analyses v.18.b^39^ in conjunction with SPM12 (http://www.fil.ion.ucl.ac.uk/spm/software/spm12/) generated connectivity analyses. When generating these connectivity matrices, additional denoising variables were generated. These denoising regressors included grey matter, white matter, CSF, and mean global signal. To generate these regressors, signal is extracted from masks generated in SPM12 by segmenting structural/T1 images into gray matter, white matter, and cerebrospinal fluid (CSF). This results in five subject-level general linear model confound regressors that are nuisance temporal components extracted from the segmented CSF and white matter, along with the motion temporal derivatives as described above. The regions of interest (ROI) used a human connectome project template atlas of intrinsic resting-state networks provided in the CONN 18.b toolbox. This network atlas includes 32 regions over 6 networks; this *a priori* network definition enabled us to test whether sleep regularity impacts network structure in a general or network specific way^4,6,36,50,51^. See Fig. 1. These template ROIs were used to generate connectivity matrices with the CONN ROI-to-ROI analyses function. In this CONN function, an ROI level time course is created by averaging voxels, and the resultant time courses are defined by Fischer’s Transformed bivariate coefficient. The result is a correlation matrix for each subject reflecting the resting-state connectivity between each ROI. Similar data acquisition and procedures were previously described in independent studies to examine self-reference and mentalization processes in individuals at clinical high risk for psychosis^52,53^.

### Graph theory analyses

ROI-to-ROI metrics (path length and clustering coefficient) were calculated using graph theory analyses, where adjacency matrix edges were weighted by cost values over 0.15 with a two-sided FDR-correction threshold of *p* < 0.05, and related to the SRI. These metrics were chosen to capture the general character of network structure among these regions^54^. Clustering coefficient describes a ratio of all possible edges to actual edges, and reflects the degree of interconnectedness of a given region within a larger network^38^. Path length is the average minimum path distance from this node to all other nodes in the network, which reflects how connected a given region is to all other regions^38^. Increased clustering coefficient or decreased path length would indicate increased network connectivity. These graph metrics were related to the SRI z-scores as the covariate of interest.

## Results

### Characteristics of the SRI in the current sample

**Figure 2:**
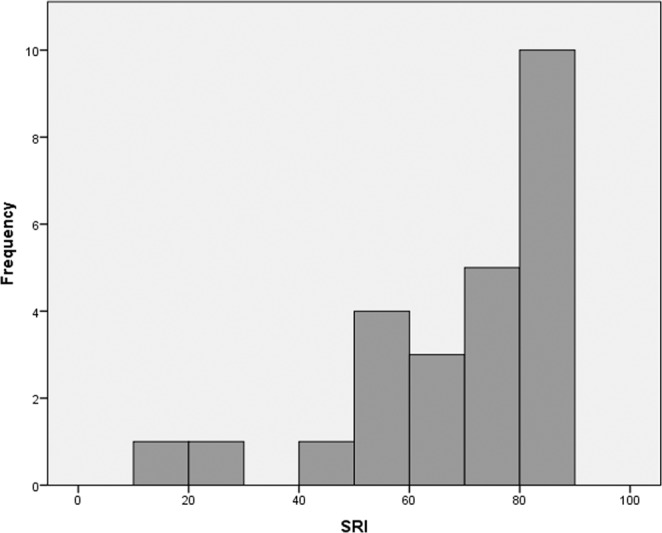
Distribution of SRI in the Current Sample. Abbreviation: SRI, Sleep Regularity Index.

The SRI in the current sample ranged from 19.68 to 88.82 (mean = 68.61, SD = 18.06) and the distribution was non-normal and negatively skewed (SW statistic = 0.86, p = 0.002). The mean and distribution of the SRI in the current study is similar to the average SRI previously reported in young adults^22^ and older adults^23^. See Fig. 2. SRI was not related to sex (t (23) = 0.99, p = 0.33), age (r = −0.002, p = 0.99), parental education (r = 0.08, p = 0.69), or race (F(2, 22) = 0.02, p = 0.99) in the current study.

### SRI and resting-state DMN path length and clustering coefficient

**Figure 3:**
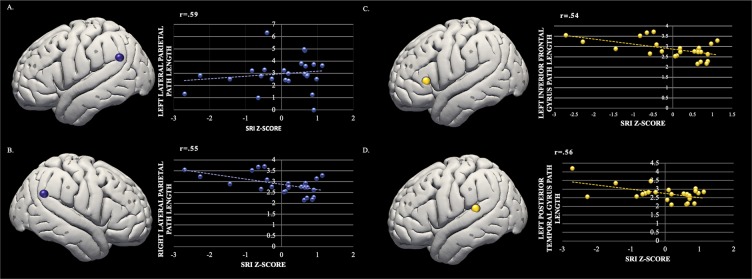
Left Panel: SRI by Path Length in the Default Mode Network including (**A**) Left Lateral Parietal and (**B**) Right Lateral Parietal. Right Panel: SRI by Path Length in the Language Network including (**A**) Left Inferior Frontal Gyrus and (**B**) Left Posterior Temporal Gyrus.

In path length analyses, increased SRI related to decreased path length (increased network connectivity) in regions within the DMN. See Table 1. Specifically, in the DMN regions, decreased path length was observed in both right and left lateral parietal lobule as SRI increased (see Fig. 3). In follow-up analyses, the addition of age and sex covariates had no impact on the direction or size of the observed effect size, nor did the addition of a total sleep time (i.e., sleep duration) covariate. In clustering coefficient analyses, there were no other significant relationships between the SRI and clustering coefficient among any DMN node (all p’s > 0.38 uncorrected).

**Table 1:** Path length statistics for associations between the SRI and significant network nodes. Abbreviations: MNI, Montreal Neurological Institute; ROI, Region of Interest.

Path Length	ROI	MNI Coordinates					Path Length Statistics		
**Default Mode Network**		*x*	*y*	*z*	*beta*	*T*	*p-uncorrected*	*p-FDR*	*post-hoc-r*
	Left Lateral Parietal	−39	−77	33	−0.99	−3.45	0.0022	0.046	0.59
	Right Lateral Parietal	47	−67	29	−0.74	−3.15	0.0045	0.046	0.55
**Language Networks**									
	Left Inferior Frontal Gyrus	−51	26	2	−0.34	−3.12	0.0049	0.046	0.30
	Left Posterior Superior Temporal Gyrus	−57	−47	15	−0.34	−3.04	0.0057	0.046	0.56

### Exploratory analyses of associations between SRI and path length and clustering coefficient within additional networks

Exploratory analyses further indicated an association between increased SRI and reduced path length (increased network connectivity) within the Language Network nodes (see Table 1). Specifically, there were two left lateralized nodes in the Language Network that significantly related to SRI including the left inferior frontal gyrus and the left posterior superior frontal gyrus (see Fig. 3). Again, the addition of age and sex covariates had no impact on the direction or size of the observed effect size, nor did the addition of a total sleep time (i.e., sleep duration) covariate. In clustering coefficient analyses, there were no other significant relationships between the SRI and clustering coefficient among any nodes or networks (all p’s > 0.12).

## Discussion

To our knowledge, this study is among the first to examine relationships between an important aspect of sleep – the regularity of sleep/wake patterns as measured by the SRI – and connectivity within a range of resting-state networks among adolescents and young adults. As the SRI is a relatively new measure, it is notable that the mean and distribution of the SRI in the current sample is similar to a prior study in young adults^22^ and was not associated with sex or age in the current sample.

Consistent with our hypothesis, we found that increased sleep regularity was associated with a more efficient network structure (decreased path length) within the DMN over and above other potential networks. Specifically, greater sleep regularity was correlated with reduced path length in the right and left lateral parietal lobule. In addition to the expected DMN network results, the present findings suggest that greater network structure efficiency of the Language Network was also associated with increased sleep regularity, including areas of the left inferior frontal gyrus and the left posterior superior frontal gyrus. Network changes were specific to path length, which may suggest that changes in sleep regularity relate to specific changes to network structure (i.e., path length rather than correlation coefficient), which has implications for the nature of previously reported reductions in network connectivity. Importantly, these associations were robust to inclusion of potential confounds including age and sex.

The current findings dovetail with a growing literature suggesting that sleep and DMN connectivity are closely linked. Specifically, this study suggests that in addition to sleep deprivation^4,6^, quality^36^, and disorders, such as insomnia and OSA^3,5,20^, greater sleep/wake irregularity may impact network structure within the DMN. Previously, reduced DMN functional connectivity has been proposed as a neural substrate of the behavioral correlates for sleep deprivation^4^ and/or may reflect a “local sleep” in which a portion of the brain enters a sleep state when an individual is otherwise conscious^7,55^. Results of the current study suggest that similar phenomena may occur for adolescents and young adults with irregular sleep patterns.

Specific reduction in the path length within the right and left lateral parietal lobule found in the present study is also consistent with prior research demonstrating abnormalities in the activity and connectivity of nodes within the inferior parietal cortices (IPC) in response to sleep complaints. Sleep deprivation is associated with reduced connectivity, spontaneous activity, and amplitude of low frequency fluctuation within IPC nodes^6,50,56^ and decreased connectivity between the IPC and additional DMN regions^50^, including the medial prefrontal cortex^4^. Additionally, deficient connectivity and spontaneous activity of the IPC has been observed in sleep-disordered populations, including insomnia^5^ and OSA^20^. Our results add to the literature suggesting that sleep deficits – in this case, irregular sleep – appear to correlate with abnormal increased path length in connecting the IPC to a larger DMN network.

The current study is among the first to examine sleep and DMN network path length during the adolescent and young adult period, a developmental stage distinguished by critical shifts in sleep, cognition, and brain structure/function^24^. Paired with a prior study, which demonstrated an association between sleep quality and resting-state DMN connectivity among adolescents^36^, these results highlight the potential importance of sleep behavior in supporting brain functioning during a crucial time of cognitive development. It is notable that the previous investigation with an adolescent sample^36^ examined variability in sleep duration and, unlike the current study, did not find an association with DMN connectivity in their sample. This may be due to the sleep regularity measure used (i.e., standard deviation in sleep duration). As mentioned above, the SRI measure has several advantages over other sleep regularity assessments in that it does not require a primary sleep period and uses a day-to-day timescale that assesses rapid changes in sleep/wake patterns^22^. Alternatively, this difference in results may reflect the nature of the metrics used, such that spontaneous activity at the voxel level may be sensitive to sleep quality, whereas sleep regularity impacts the path length at particular places in the DMN network without impacting the global or correlation coefficient between these regions. Thus, the SRI may be particularly sensitive to assessing irregularity in sleep patterns relating to poorer DMN network structure.

Results of the current study suggest several exciting directions for future research. First, given the prominent role of the DMN in supporting aspects of cognition, including internally-oriented, self-referential processes^10,12–15^, future research should examine whether the DMN mediates relationships between irregular sleep and poorer cognition observed during adolescence and young adulthood^21^. Second, as both reduced DMN connectivity and sleep complaints are often observed among adolescents with neuropsychiatric conditions, such as ADHD, depression, and emerging psychosis^17,18,57–60^, future studies should examine how the interplay between sleep regularity and DMN connectivity and network structure impacts the presentation of these disorders during this developmental period. Indeed, a recent study has shown that poor sleep quality is related to increased impulsivity among adolescents with low, but not high, connectivity between the DMN and prefrontal cortex, suggesting DMN connectivity may contribute to links between sleep and behavioral outcomes in this age group^61^. Additionally, another recent investigation has suggested that greater sleep irregularity, as measured by the SRI, may mediate the relationship between delayed sleep onset time and daytime functioning in work/school, social, and family domains^62^.

Finally, exploratory analysis in the current study indicated a relationship between sleep regularity and network structure efficiency within the Language Network, particularly in areas of the left inferior frontal gyrus and the left posterior superior frontal gyrus. Relative to the DMN, very little is known about how sleep may relate to connectivity within this network. However, it is notable that from a behavioral perspective, sleep irregularity in general has been linked to poorer verbal cognition in youth^21^ and the SRI specifically is associated with poorer communication among youth with Autism^63^. Future investigations using larger samples of adolescents and young adults should replicate and further elucidate a possible link between sleep regularity and the Language Network.

This study should be interpreted in the context of its limitations. First, this study assessed the SRI and resting-state connectivity at a single time point. As such, direction of causation between irregular sleep and reduced DMN connectivity cannot be inferred. Second, this study utilized a relatively small sample spanning the adolescent and young adult period which does not allow for investigation of how relationships between sleep regularity and DMN connectivity may change over the course of development. Future studies using larger samples and longitudinal and/or experimental designs may shed light on these questions. Third, MRI scans in the current study were scheduled to accommodate adolescent and young adult schedules and as such the timing of scans were not standardized. As prior work using experimental sleep deprivation paradigms have shown that sleep pressure may impact DMN functional connectivity^4^, future studies controlling for time awake prior to the scan may assess the relative impact of sleep regularity versus sleep pressure on DMN connectivity.

In conclusion, irregular sleep patterns as measured by the SRI are associated with increased path length within the DMN – specifically in the right and left lateral parietal lobule – among adolescents and young adults, suggesting that sleep regularity may be vital for optimal brain functioning during this developmental period. Future studies may assess how this relationship impacts cognitive development and performance as well as neuropsychiatric outcomes in this age group. If sleep irregularity is found to impact neurocognitive performance and disorders through alterations in brain connectivity, sleep stabilization techniques, such as setting consistent bed- and wake-times^64^, may prove beneficial for adolescents’ cognitive and brain development as well as neuropsychiatric health.

## Data Availability

The datasets generated during and/or analyzed during the current study are available from the senior author on reasonable request.
